# Impaired Empathy Processing in Individuals with Internet Addiction Disorder: An Event-Related Potential Study

**DOI:** 10.3389/fnhum.2017.00498

**Published:** 2017-10-10

**Authors:** Can Jiao, Ting Wang, Xiaozhe Peng, Fang Cui

**Affiliations:** ^1^College of Psychology and Sociology, Shenzhen University, Shenzhen, China; ^2^Shenzhen Key Laboratory of Affective and Social Cognitive Science, Shenzhen University, Shenzhen, China

**Keywords:** internet addiction disorder (IAD), empathy, ERPs, N2, P3

## Abstract

Internet addiction disorder (IAD) is associated with deficits in social communication and avoidance of social contact. It has been hypothesized that people with IAD may have an impaired capacity for empathy. The purpose of the current study was to examine the processing of empathy for others’ pain in IADs. Event-related potentials produced in response to pictures showing others in painful and non-painful situations were recorded in 16 IAD subjects and 16 healthy controls (HCs). The N1, P2, N2, P3, and late positive potential components were compared between the two groups. Robust picture × group interactions were observed for N2 and P3. The painful pictures elicited larger N2 and P3 amplitudes than the non-painful pictures did only in the HC group but not in the IAD group. The results of this study suggest that both of the early automatic and of the later cognitive processes of pain empathy may be impaired in IADs. This study provides psychophysical evidence of empathy deficits in association with IAD. Further studies combining multidimensional measurements of empathy are needed to confirm these findings.

## Introduction

Internet addiction disorder (IAD) has been described as an inability to control internet use despite severe negative consequences and is generally conceptualized as a behavioral addiction (Tam and Walter, 2013; D’Hondt et al., 2015; Kuss and Lopez-Fernandez, 2016), representing a specific impairment that involves online and/or offline web misuse, and it is mainly relevant for young generations (Grant et al., 2010; Balconi et al., 2017). Although whether IAD is a mental disorder *per se* is still controversial, the public health and social issues related to IAD are clear and the neural correlates of IAD have begun to be explored (D’Hondt and Maurage, 2015).

It has been suggested that IAD may have some commonalities with substance abuses. For example, individuals with IAD show a decreased executive control ability, reflecting in the lack of ability to inhibit the behavior once it has been initiated or to refrain from the behavior after a period of abstinence (Brand et al., 2014; D’Hondt and Maurage, 2015). Electrophysiological studies have shown reduced N2 amplitudes in the NoGo trials in a Go/NoGo task as well as a reduced medial frontal negativity (MFN) amplitude in the incongruent trials of the Stroop task, findings which are suggestive of an impairment of executive control (Dong et al., 2011). Besides, individuals with IAD also show impaired processing of social stimuli, such as faces. He et al. (2011) found that compared to healthy controls (HCs), subjects with IADs showed reduced P1 and N170 in the processing of faces.

Empathy refers to the ability to share and understand others’ emotions or feelings (Decety and Lamm, 2006). Experiencing empathy relies on the integration of two components: an automatic early emotional contagion system and a higher-level cognitive system that allows the self-regulation and elaboration of the situations (Decety and Jackson, 2004; Mella et al., 2012). The ability to empathize with others has long been viewed as crucial for successful social interactions (Hetu et al., 2012). The amount of internet use has been found to correlate inversely with the ability to interact with other people (Engelberg and Sjoberg, 2004). Accordingly, people with IAD often neglect their social lives (Young, 1998a). Hence, empathy may be an influential factor in the progressive diminution of real-life social interactions among internet users (Melchers et al., 2015).

However, studies focusing on the neural basis of empathic processing in IADs are still very rare. Thus far, to our knowledge, only two studies have investigated empathy in IADs. Firstly, Melchers et al. (2015) obtained evidence of a negative relationship between internet addiction and empathy, as evidenced by self-reported empathy and problematic internet use scores (Melchers et al., 2015). Secondly, an event-related potential (ERP) study found that youth with IAD showed a reduced difference in N2 amplitudes between painful and non-painful stimuli comparing to HCs when processing pictures showing others in pain (Wang et al., 2014). The first aforementioned study was an exploratory correlation study and the second one involved a very specific population (Chinese urban left-behind children), limiting its generalizability.

Empathy for pain has been shown to involve two distinct temporally processes. The first is an early automatic, bottom-up process, reflected by the N1, P2, and N2 components that correspond to emotional contagion and affective sharing. Secondly, there is a top-down controlled, cognitive process, reflected by the P3 and late positive potential (LPP) components that regulates empathic responses and makes a clear self-other distinction (Fan and Han, 2008; Mella et al., 2012; Sessa et al., 2014). The bottom-up process refers to the unconscious and automatic emotional contagion and affective sharing triggered involuntarily by observing other’s pain, which is not influenced by instructions or task demands. The top-down controlled process, on the other hand, refers to the process that is under control of the intentions of the observer and can be voluntarily modulated by factors such as the instructions, task demands, prior experience, social relations, etc., (Fan and Han, 2008). Thus, this model can help us to resolve which stage of empathy may be impaired in IADs.

IAD may be comorbid with other psychiatric states, especially depression and anxiety (Sanders et al., 2000; Yen et al., 2007; Wei et al., 2012; Lai et al., 2015). Depressed individuals show reduced awareness of others’ emotion, impaired emotion recognition, and deficits in empathy and perspective taking [for review, (Kupferberg et al., 2016)]. Anxiety can also reduce affective empathic responses to others’ pain (Negd et al., 2011). Hence, comorbid depression and anxiety may be influential confounding factors in the present study. Thus, we employed exclusion criteria for detection of signs of depression or anxiety.

The aim of the current study was to explore how the processing of others’ pain may be different among individuals with IAD and HCs. We hypothesized that the IADs would be less responsive, or less discriminative to other’s pain than the HCs. If the early automatic stage of empathy is impaired, it should be evident in the N1 (Ibanez et al., 2011; Lyu et al., 2014), P2 (Rutgen et al., 2015), and/or N2 (Cui et al., 2016a) components. Conversely, if the voluntary, top-down processing is impaired, then it should be evident in the P3 and/or LPP (Ibanez et al., 2011).

## Materials and Methods

### Ethics Statement

All research procedures were approved by the Medical Ethical Committee of Shenzhen University Medical School according to the Declaration of Helsinki. All participants were given written informed consent after they fully understand the study.

### Participants

A total number of 16 participants with IAD and 16 HCs were recruited from local universities. There was no significant difference between the two groups with respect to age, handedness, and education. We used Young’s Internet Addiction Test (IAT) to screen for IAD (Young, 1998b). All IAD subjects were with a score of ≥ 40 on the IAT (Note: IAT scores on 40–60 indicate mild internet addiction; 60–80 indicate moderate internet addiction; and 80–100 indicate server internet addiction). Moreover, because IAD may be comorbid with other psychiatric states, especially depression and anxiety, we excluded IAD participants who scored ≥ 40 on either the Zung Self-Rating Depression Scale (SDS) (Zung, 1965) or the Zung Self-Rating Anxiety Scale (SAS) (Zung, 1971) (the cutoff scores are 53 for SDS and 50 for SAD in Chinese norm). Exclusion criteria for both IAD and control participants were as follows: pregnancy, history of head injury, and other neurological disorders substance abuse or dependence in past 6 months.

### Stimuli

**FIGURE 1:**
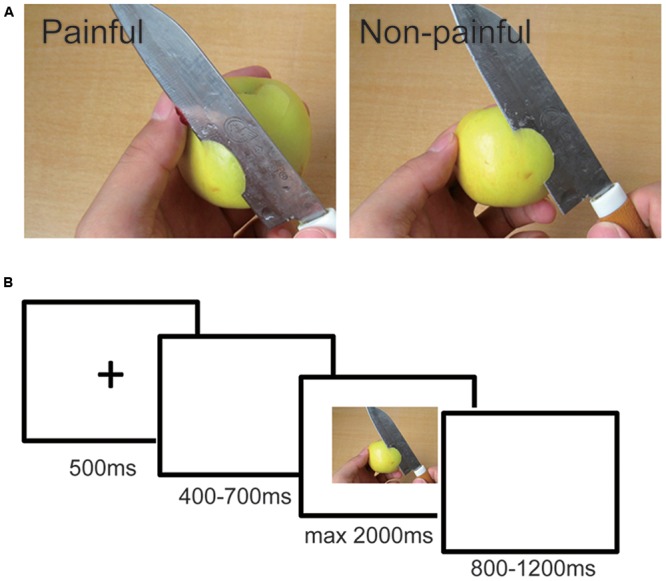
**(A)** Examples of the pictures (Left panel: painful picture; Right panel: non-painful picture); **(B)** Structure of one trial. Each trial started with a 500 ms fixation, after a 400–700 ms random blank interval, the picture appeared for a maximum of 2000 ms and the participants were supposed to judge if the picture was a painful one or a non-painful one as soon and accurately as possible. The picture disappeared when a response was given. The ISI between trials was 800–1200 ms randomly.

The visual stimuli used were pictures showing a person’s hands/forearms/feet in painful or non-painful situations, which have been used in previous ERP studies (Meng et al., 2012; Meng et al., 2013). All the situations depicted in these pictures were ordinary events in daily life. All the events showing in the non-painful pictures were corresponding to those in the painful pictures, but without the nociceptive component (**Figure 1A**). There were 60 painful pictures and 60 non-painful pictures in total. All of them had the same size of 9 × 6.76 cm (width × height) and 100 pixels per inch. Luminance, contrast, and color were matched between painful and non-painful pictures. Previous studies have confirmed that painful and non-painful pictures were significantly different on the dimensions of pain intensity, arousal level, and emotional valence, according to self-reported rating (Meng et al., 2012).

### Experimental Procedures

Stimulus display and behavioral data acquisition were conducted using E-Prime software (Version 2.0, Psychology Software Tools, Inc., Boston, MA, United States). During the task, participants sat comfortably in an electrically shielded room approximately 90 cm from a 15-inch color computer screen.

The participants were asked to observe pictures. In each trial, a fixation was presented on a white screen for 500 ms, followed a 400- to 700-ms blank interval. Then the target picture would be presented for a maximum of 2000 ms. The ISI between trials was 800–1200 ms randomly (**Figure 1B**). Participants were instructed to judge whether this picture showing a painful or non-painful situation as quickly as possible by pressing “F” or “J” buttons on the keyboard placed in front of them. The button pressing was counter-balanced among participants. The picture would disappear as soon as a response was given. There are four sessions in the experiment. Each session contains 60 trials, including 30 painful pictures and 30 non-painful pictures. Each picture repeated twice in total. Four conditions were generated accordingly: IADs observing the painful pictures (*IAD_P*); IADs observing the non-painful pictures (*IAD_NP*); HCs observing the painful pictures (*HC_P*); and HCs observing the non-painful pictures (*HC_NP*). After the task, both groups of participants were asked to fulfill the Interpersonal Reactivity Index (IRI). The IRI has been one of the most widely used indices of empathy, which is a questionnaire that assesses the empathy trait using four subscales: perspective taking, fantasy, empathic concern, and personal distress (PD) (Davis, 1983).

### EEG Acquisition and Preprocessings

Electroencephalography (EEG) data were recorded from a 63-electrodes scalp cap using the 10–20 system (Brain Products, Munich, Germany). The channel TP10 was used as the reference during recording. Two electrodes located above and below the left eye were used to measure the electrooculogram (EOG). EEG and EOG activities were amplified at 0.01–100 Hz band-passes and sampled at 500 Hz. All electrode impedances were maintained below 5 kΩ.

Electroencephalography (EEG) data were preprocessed and analyzed using MATLAB R2011b (MathWorks) and EEGLAB toolbox (Delorme and Makeig, 2004). EEG data at each electrode were re-referenced to the average of the left and right mastoids before further analysis. Then the signal passed with 0.01–30 Hz band-pass filter. Time windows of 200 ms before and 1000 ms after the onset of picture stimuli were segmented from EEG and the whole epoch was baseline-corrected by the 200 ms prior to the onset of the picture. EOG artifacts were corrected using an independent component analysis (ICA) (Jung et al., 2001). Epochs with amplitude values exceeding ± 50 μV at any electrode were excluded from the average, and all trials with incorrect responses were excluded from further analysis [Rejected epochs: 16.75 ± 6.04 (HCs); 18.25 ± 2.35 (IADs)].

### Statistics

For the reaction time and accuracy, a two-way repeated-measures ANOVA was performed with pictures (painful picture and non-painful picture) as the within-subject factor and groups (IAD and HC) as the between-subject factor. Descriptive data were presented as the (mean ± SE). The significance level was set at *p* < 0.05.

This study focused on the ERPs elicited by pictures showing others in painful and non-painful situations. We analyzed the components of the frontal N1 (90–150 ms), frontal P2 (180–220 ms), fronto-central N2 (200–280 ms), parietal P3 (300–400 ms), and centro-parietal LPP (550–650 ms) according to grand-averaged ERP, the topographies, and relevant literature (Decety et al., 2010; Meng et al., 2013). Mean amplitudes were measured for each component. Notice that the time windows selected were mainly based on the grand-averaged ERP of all trials for painful and non-painful conditions from both groups. The time windows of several components were slightly different from the literature [40–50 ms before or after the time windows selected in the previous papers (Meng et al., 2012, 2013)]. To demonstrate that the results were not artifacts of the prior selected time windows, we conducted the following-up analyses using varying time windows (move forward 40 ms and move backward 40 ms) for the components that reported significance. All of the results revealed a similar pattern of results (we reported the results from the time window in the middle). By conducting analyses using time windows of varying duration/onset/offset, it could show that the significance of the results is a consistent effect (Bacigalupo and Luck, 2015; Luck and Gaspelin, 2017).

Further statistical analysis was conducted in IBM SPSS Statistics 22 (IBM Corp., Armonk, NY, United States). Previous studies using similar stimuli suggested the early component N1, P2, N2, and the late components P3, LPP were particularly related to observing other’s pain. On the basis of the topographical distribution of grand-averaged ERP activity and the previous studies, different sets of electrodes for each component were chosen (Meng et al., 2012, 2013; Lyu et al., 2014). F3, Fz, F4, FC3, FCz, and FC3 were selected for the analysis of N1 and N2; FC3, FCz, FC4, C3, Cz, and C4 were selected for the analysis of P2; CP3, CPz, CP4, P3, Pz, and P4 were selected for the analysis of P3; C3, Cz, C4, CP3, CPz, and CP4 were selected for the analysis of LPP. Repeated-measures ANOVA with pictures (painful and non-painful) as the within-subject factor and groups (IAD and HC) as the between-subject factor were performed for the mean amplitudes of all selected electrodes sites for each component. All statistical analyses met the requirements of parametrical statistical tests. Degrees of freedom for F-ratios were corrected according to the Greenhouse–Geisser method. Statistical differences were considered significant at *p* < 0.05; *post hoc* comparisons were Bonferroni corrected at *p* < 0.05.

To examine whether the effect we observed in ERP data was related to the participant’s empathic trait, firstly, we calculated the differences between the amplitudes of ERPs elicited by the painful stimuli and the non-painful stimuli in the time windows of N2 and P3. The amplitudes were calculated as the mean of the amplitudes of all selected electrodes (F3, Fz, F4, FC3, FCz, and FC3 for N2; CP3, CPz, CP4, P3, Pz, and P4 for P3). Secondly, we run Pearson correlation analyses between the differences of ERP amplitudes and the scores of the four subscales of IRI, separately.

## Results

### Behaviors

For the accuracy rate, the main effect of picture [*F*(1,30) = 1.854, *p* = 0.183, $\eta_{p}^{2}$ = 0.058), group (*F*(1,30) = 0.557, *p* = 0.461, $\eta_{p}^{2}$ = 0.018], and the interaction of picture × group [*F*(1,30) = 0.146, *p* = 0.705, $\eta_{p}^{2}$ = 0.005] were not significant (range of accuracy rate: 79–99%, mean ± SE: 91.25 ± 4.8%). For reaction time, we found a significant main effect of picture [*F*(1,30) = 23.662, *p* < 0.001, $\eta_{p}^{2}$ = 0.441]. Both groups response faster to painful situation comparing to the non-painful situation (*IAD_P*: 633.488 ± 54.928 ms; *IAD_NP*: 669.714 ± 74.255 ms; *HC_P*: 645.528 ± 55.207 ms; *HC_NP*: 684.085 ± 61.851 ms). The main effect of group [*F*(1,30) = 0.413, *p* = 0.525, $\eta_{p}^{2}$ = 0.014] and the interaction of picture × group [*F*(1,30) = 0.023, *p* = 0.880, $\eta_{p}^{2}$ = 0.001] were not significant (range of RTs: 554–861 ms; mean ± SE: 659.5 ± 62.6 ms).

For IRI scores, we run independent *t*-tests to compare the scores of IAD group and HC group for all four subscales. It was found that on the subscale “PD,” the scores of the IAD group were significantly smaller than the scores of the HC group [IAD: 8.125 ± 0.875; HC: 10.375 ± 0.651; *t*(30) = -2.063, *p* = 0.048]. The differences between two groups of the other three subscales were not significant (*p* > 0.116) (**Table 1**).

**Table 1:** Participants demographics for IAD participants and healthy controls.

Items	IAD (*n* = 16)	Control (*n* = 16)	Statistics
Age(y)	19.94 ± 1.289 (18–23)	20.75 ± 1.437 (19–24)	*t* (30) = -1.638, *p* = 0.103
Gender (male/female)	12/4	13/3	
Education time (y)	15.625 ± 0.375 (12–19)	15.750 ± 0.403 (12–19)	*t* (30) = -0.227, *p* = 0.822
Handedness (Right/Left)	15/1	15/1	
IAT score	58.125 ± 2.809 (49–78)	33.875 ± 1.224 (23–36)	*t* (30) = 7.914, *p* < 0.001
SAS	30.625 ± 1.309 (23–38)	27.000 ± 1.144 (21–35)	*t* (30) = 2.085, *p* = 0.064
SDS	33.937 ± 0.844 (30–39)	32.500 ± 0.966 (27–39)	*t* (30) = 1.121, *p* = 0.271
IRI Perspective Taking	11.750 ± 0.715 (6–16)	13.188 ± 0.526 (8–16)	*t* (30) = -1.618, *p* = 0.116
Empathic Concern	14.375 ± 1.281 (3–23)	14.312 ± 0.930 (8–22)	*t* (30) = 0.39, *p* = 0.969
Personal Distress	8.125 ± 0.875 (1–14)	10.375 ± 0.651 (6–15)	*t* (30) = -2.063, *p* = 0.048
Fantasy	15.56 ± 1.201 (4–23)	16.312 ± 0.889 (9–20)	*t* (30) = 0.792, *p* = 0.619

*Descriptive data are presented as mean ± SE (range). IAT, Internet Addiction Test; SAS, Zung Self-Rating Anxiety Scale; SDS, Zung Self-Rating Depression Scale; IRI, Interpersonal Reactivity Index*.

### Event-Related Potentials (ERPs)

*N1.* The main effect of picture [*F*(1,30) = 3.180, *p* = 0.085, $\eta_{p}^{2}$ = 0.096], the main effect of group [*F*(1,30) = 0.465, *p* = 0.500, $\eta_{p}^{2}$ = 0.015] and the interaction of picture × group [*F*(1,30) = 0.131, *p* = 0.720, $\eta_{p}^{2}$ = 0.004] were not significant.

*P2.* The main effect of picture [*F*(1,30) = 1.550, *p* = 0.223, $\eta_{p}^{2}$ = 0.049], the main effect of group [*F*(1,30) = 0.098, *p* = 0.756, $\eta_{p}^{2}$ = 0.003] and the interaction of picture × group [*F*(1,30) = 0.729, *p* = 0.400, $\eta_{p}^{2}$ = 0.024] were not significant.

**FIGURE 2:**
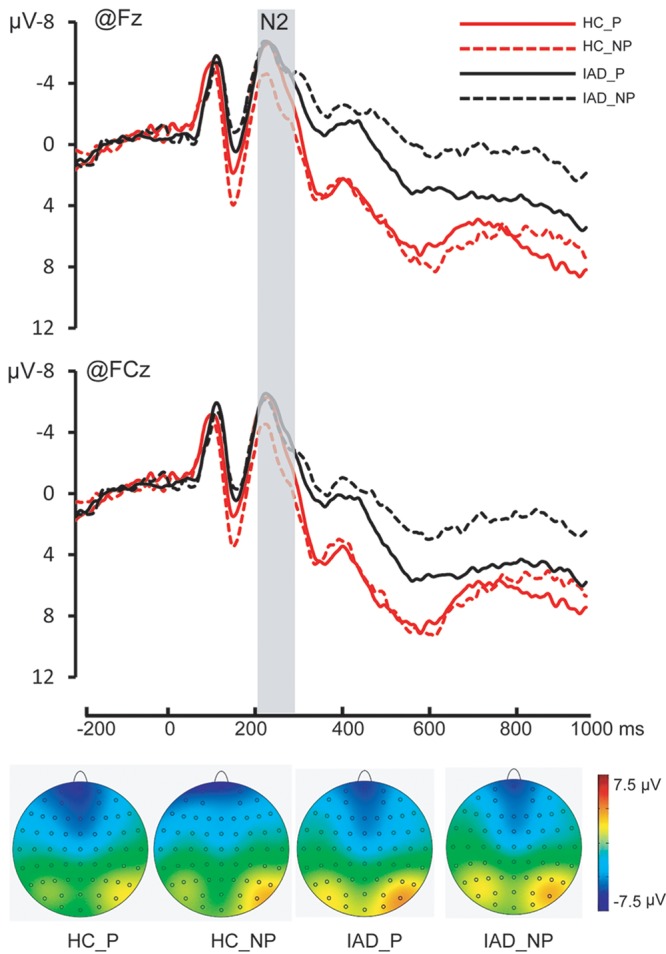
The Grand average on Fz and FCz and topography of N2 in all four conditions [Painful pictures in HC group (HC_P); Non-painful pictures in HC group (HC_NP); Painful pictures in IAD group (IAD_P); and Non-painful pictures in IAD group (IAD_NP)]. The time window of the topography was corresponding to the gray square covered area.

*N2*. The main effect of picture was significant [*F*(1,30) = 6.406, *p* = 0.017, $\eta_{p}^{2}$ = 0.176]. Painful pictures elicited significantly more negative amplitudes than the non-painful pictures (-6.301 ± 0.745 μV and -5.650 ± 0.769 μV). The main effect of group was not significant [*F*(1,30) = 0.039, *p* = 0.845, $\eta_{p}^{2}$ = 0.001]. The interaction of group × picture was significant [*F*(1,30) = 6.838, *p* = 0.016, $\eta_{p}^{2}$ = 0.177]. Pairwise comparisons showed that the amplitudes elicited by the painful pictures were significantly more negative than the amplitudes elicited by the non-painful pictures only in the HC group (-6.481 ± 1.088 μV and -5.176 ± 1.054 μV, *p* = 0.001) but not in the IAD group (-6.124 ± 1.088 μV and -6.122 ± 1.054 μV, *p* = 0.577) (**Figures 2**, **4A** and **Table 2**).

**FIGURE 3:**
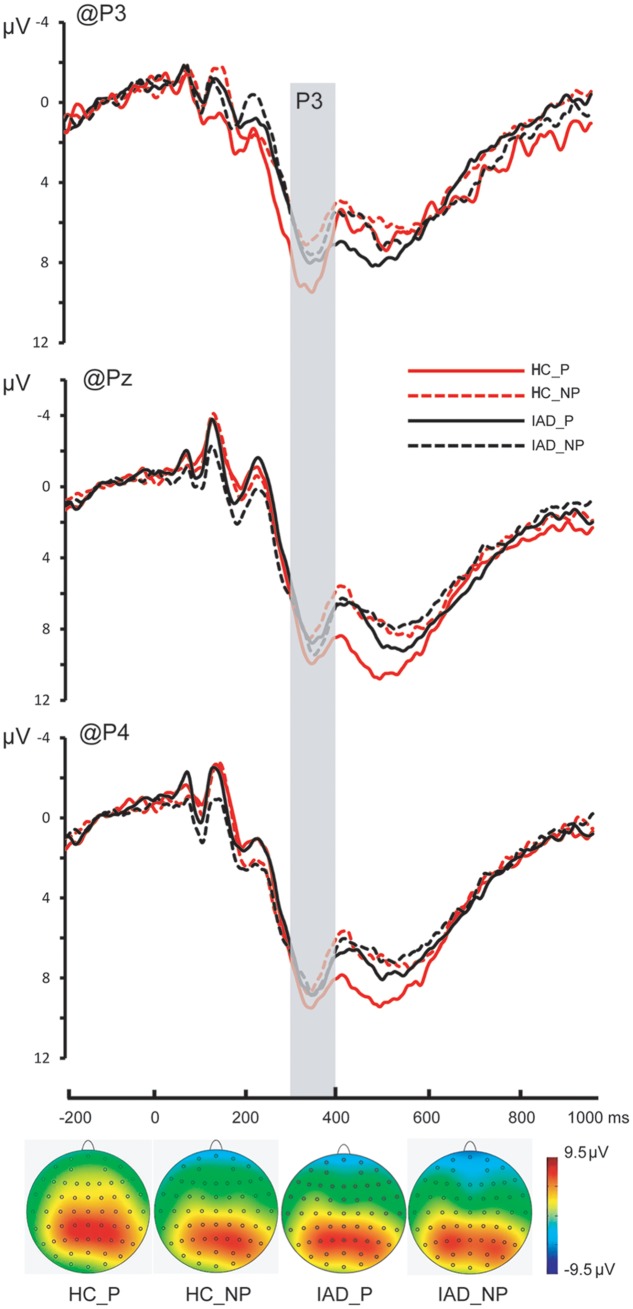
The Grand average on P3, Pz, and P4; the topography of P3 in all four conditions (the time window of the topography was corresponding to the gray square covered area).

**FIGURE 4:**
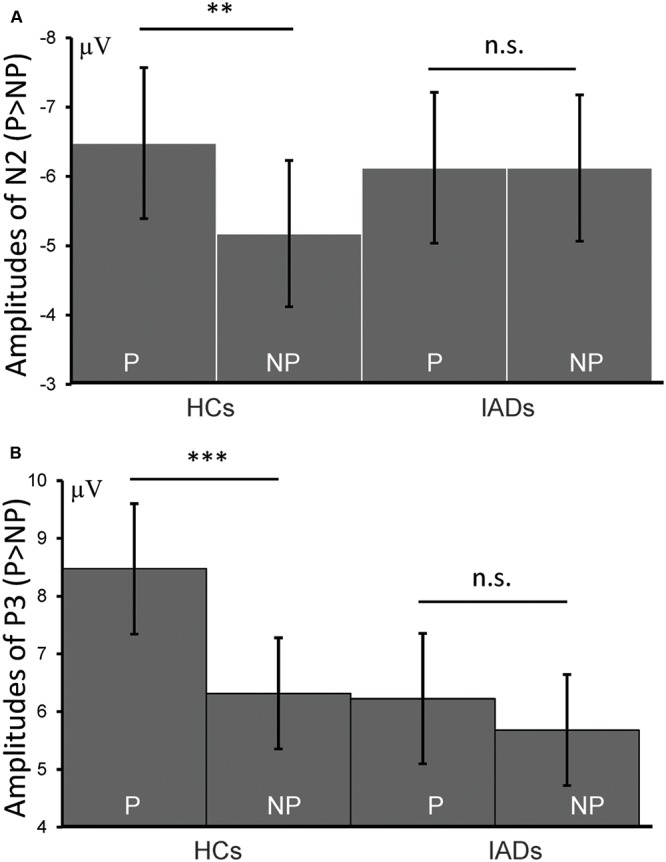
Interactions of picture × group on N2 **(A)** and P **(B)** (^∗∗∗^*p* < 0.001; ^∗∗^*p* < 0.01; n.s., not significant).

**Table 2:** Descriptive statistics for event-related potential (ERP) data.

Group	Picture	N1(μV)	P2(μV)	N2(μV)	P3(μV)	LPP(μV)
HC	P	-6.605 ± 0.842	2.805 ± 1.030	-6.481 ± 1.088	8.473 ± 1.130	8.542 ± 1.033
	NP	-6.197 ± 0.846	2.572 ± 0.887	-5.176 ± 1.054	6.316 ± 0.961	7.313 ± 1.019
IAD	P	-6.310 ± 0.756	2.874 ± 1.092	-6.124 ± 1.088	6.227 ± 1.130	7.299 ± 1.209
	NP	-5.899 ± 0.961	2.158 ± 0.941	-6.122 ± 1.054	5.681 ± 0.961	4.7995 ± 0.890

*HC: healthy control; IAD: Internet Addiction Disorder; P: painful; NP: non-painful; LPP: late positive potential*.

*P3*. The main effect of picture was significant [*F*(1,30) = 17.668, *p* < 0.001, η_*p*_^2^ = 0.3371]. Painful pictures elicited significantly larger amplitudes than the non-painful pictures (7.350 ± 0.799 μV and 5.998 ± 0.679 μV). The main effect of group was not significant [*F*(1,30) = 0.989, *p* = 0.328, $\eta_{p}^{2}$ = 0.032]. The interaction of group × picture was significant [*F*(1,30) = 6.283, *p* = 0.018, $\eta_{p}^{2}$ = 0.173]. Pairwise comparisons showed that the difference between the painful and the non-painful pictures was only significant in the HC group (8.473 ± 1.130 μV and 6.316 ± 0.961 μV*, p* < 0.001) but not in the IAD group (6.227 ± 1.130 μV and 5.681 ± 0.961 μV*, p* = 0.240) (**Figures 3**, **4B** and **Table 2**).

*LPP.* The main effect of picture was significant [*F*(1,30) = 22.517, *p* < 0.001, $\eta_{p}^{2}$ = 0.429]. Painful pictures elicited significantly larger amplitudes than non-painful pictures (7.469 ± 0.761 μV and 5.787 ± 0.674 μV). The main effect of group [*F*(1,30) = 1.128, *p* = 0.297, $\eta_{p}^{2}$ = 0.036] and the interaction of picture × group [*F*(1,30) = 2.055, *p* = 0.162, $\eta_{p}^{2}$ = 0.064] were not significant.

### Subjective Reports and Their Correlations with Neural Activity

**FIGURE 5:**
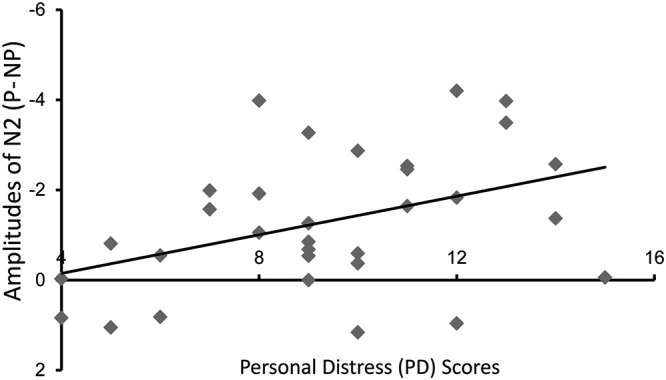
Correlation between amplitudes of N2 (painful > non-painful) and PD scores.

Results of the correlation analyses showed that the difference of N2 (painful and non-painful) was significantly correlated with the scores of the “PD” of the IRI [*r* (30) = -0.407, *p* = 0.021] (**Figure 5**).

## Discussion

The present study explored the neural underpinnings of empathic responses to other’s pain in the IADs. The IAD group was found to be less discriminative than the HC group to other’s pain in both of the early automatic and of the later cognitive controlled processing stages, supporting by the ERP data. These results are consistent with the suggestion that IAD is associated with empathy deficit (Melchers et al., 2015).

It should be noticed that in the literature of ERP studies focusing on empathy for pain, there were studies that reported a positive shift of the painful condition comparing to the non-painful condition (Fan and Han, 2008; Sheng and Han, 2012). There were other studies reported an insignificant result in the early components, and the positive shift was only observed in the later components such as P3 and LPP (Meng et al., 2013). Besides, there are also studies reported a more negative shift in the early components and a more positive shift in the later components (Cui et al., 2016a,b). This inconsistency implies that only using the amplitudes of ERP components to indicate the neural responses was unstable. We proposed to use the discrimination between the painful and non-painful stimuli to indicate how well the stimuli were processed. If the painful and non-painful stimuli were differentiated under one condition but not in the other condition, we can say the stimuli was better processed in the former one. This logic has been applied in the literature (Ibanez et al., 2011; Cui et al., 2016a,b).

The early component N1 has been shown to discriminate painful from non-painful stimuli and has been described as an index of automatic activation of affective arousal (Lyu et al., 2014). Some studies have reported that observing other’s pain induced a more positive N1 component than the non-painful stimuli (Fan and Han, 2008; Han et al., 2008; Decety et al., 2010; Ibanez et al., 2011), whereas others reported no effect of observing others’ pain on N1 amplitude (Mella et al., 2012; Lyu et al., 2014). This inconsistency across studies may be due to methodological differences, such as different sets of stimuli. However, these inconsistent findings also suggest the effect of the pictures on N1 was not stable and can be easily influenced by contextual factors. In the current study, we did not find significant differences in N1 in response to viewing painful versus non-painful pictures in either the IAD or the HC group.

The N2 component has been suggested to reflect the early automatic sensitivity to other’s pain (Chen et al., 2012). The amplitude of N2 has been reported to correlate with the subjective ratings of affective empathy and Empathic Concern Scale scores (Sessa et al., 2014). Interestingly, we observed a significant group × picture interaction effect on N2, wherein a difference in painful versus non-painful image stimulus was observed in the HCs, but not in the IADs. This finding suggests that the individuals with IAD may have a reduced sensitivity to others’ pain, in terms of elicitation of affective arousal and emotional sharing.

In addition, we found that the difference of N2 evoked by painful and non-painful pictures was significantly correlated with the scores in the PD subscale of IRI. The larger the difference between painful and non-painful conditions was the higher PD score the participant had. The PD scale was designed to measure the discomfort generated in response to observing others in pain. Previous studies suggested that the automatic affective sharing with other’s emotional experience may lead to PD (Preston and de Waal, 2002; Gallese, 2003; Lamm et al., 2007). This significance correlation suggested that the discrimination between painful and non-painful stimuli in the time window of N2 reflected the level of discomfort induced by affective sharing with other’s pain. Besides, when comparing the IRI score between the two groups, the only significant difference was that the scores of PD: the HCs’ scores were significantly higher than the IADs’ scores. This result also supported that the affective sharing with other’s pain was different in the two groups.

We observed a similar group × picture interaction on the P3 component wherein greater P3 amplitude was triggered in response to viewing painful pictures than non-painful pictures only in the HC group, but not in the IAD group. P3 amplitude has been associated with motivational significance, arousal level, and the influence of these factors on mental resource allocation (Olofsson et al., 2008). Generally, highly salient, arousing, or motivational stimuli elicit larger P3 (Delplanque et al., 2004; Nieuwenhuis et al., 2005). It is found that P3 amplitudes elicited in the physicians were relatively insensitive to the distinction between painful and non-painful stimuli compared to other non-physician control participants, perhaps due to physician habituation (Decety et al., 2010). A similar P3 insensitivity in the IAD group suggested that individuals with IAD may allocate less attentional resources to the processing of other’s pain and may be less emotionally involved with other’s pain.

Besides, it was worthy to mention that the results presented here do not necessarily indicate a causal relationship between the empathic deficits and IAD. Does the addiction to internet result in the lack of empathy or the persons lacking empathy is more vulnerable to addiction? As suggested by a recent review and a few studies, empathy might have a protective function in resisting addictions (Massey et al., 2017). For example, one study found that a greater capacity to recognize facial expressions of sadness, anger, and fear in others were independently associated with a lower likelihood of smoking during pregnancy for women with a genetic predisposition to the sensitivity of social context (Massey et al., 2015). Children with a severe deficit in affective empathy may be at increased risk for early substance use (Frick and White, 2008; Swendsen et al., 2010). Moreover, in the IAD population, the number of males was significantly higher than that of females while females reported having a significantly higher level of empathy than males (Han et al., 2008; Jang and Ji, 2012; Becker et al., 2017). As such, the present study only determines the existence of empathic deficits in IADs but more longitudinal studies are needed to determine the causal relationship between empathy and IADs.

In conclusion, the current findings suggested that IADs showed reduced sensitivity to other’s pain. Specifically, the reduced painful versus non-painful image stimulus differences in N2 and P3 amplitude in IADs, relative to HCs, suggest that they have reduced affective arousal and emotional sharing as well as the allocation of attentional resources to others’ pain, respectively. These findings may help to elucidate the impaired social functioning observed in IADs.

### Limitations

One limitation of the present study was that there were no subjective measures of social deficits. Although the ERP index support that IADs are less discriminative than HCs to other’s pain, the lack of behavioral measurement weakens our argument. This lack of significance in behavioral data may due to the small sample sets (*n* = 16 in each group). More subjective measures of empathic ability or larger sample set should be collected in further researches. For example, instead of simply asking the participants to judge whether the picture is a painful one, we can ask them to rate how painful the person feels or how unpleasant other’s pain makes them feel. The correlations between these subjective measurements and ERP index can better associate the finding of neural activities to behavioral deficits.

## Author Contributions

FC designed the experiment. CJ and TW collected and analyzed the data. CJ, TW, and XP wrote the main manuscript. FC and CJ prepared figures. All authors reviewed the manuscript.

## Conflict of Interest Statement

The authors declare that the research was conducted in the absence of any commercial or financial relationships that could be construed as a potential conflict of interest.
